# T-S fuzzy approach for real-time vehicle state estimation and road safety enhancement

**DOI:** 10.1038/s41598-025-19076-6

**Published:** 2025-10-08

**Authors:** Mohamed Saber, Mohamed Ouahi, Saad Motahhir, Abdelhamid Rabhi, Nabil El Akchioui

**Affiliations:** 1 Laboratory of Applied Sciences and Emerging Technologies, National School of Applied Sciences, BP 72, My Abdallah Avenue Km. 5 Imouzzer Road, Fez, Morocco; 2 Laboratory of Applied Sciences and Innovative Technologies, National School of Applied Sciences, BP 72, My Abdallah Avenue Km. 5 Imouzzer Road, Fez, Morocco; 3 MIS Laboratory, University of Picardie Jules Verne, 80025 Amiens, France; 4 Laboratory of R&D in Engineering Sciences, Faculty of Science and Technology Al Hoceima, Abdelmalek Essaadi University, Tangier-Tetouan-Al Hoceima, Morocco

**Keywords:** Vehicle Dynamics, Functional Observer, T-S Fuzzy Model, LMI, Unknown Input Estimation, Engineering, Mechanical engineering

## Abstract

Safe driving requires a comprehension of critical instances and dangers, achievable only through accurate estimation of dynamic vehicle behavior and road characteristics. This study proposes a Takagi-Sugeno (T-S) fuzzy functional observer capable of real-time estimation of unmeasured states (side-slip angle, yaw rate, angular displacement) and unknown inputs such as road curvature, using Lyapunov-Krasovskii stability theory and Linear Matrix Inequalities (LMIs) for parameter design. The objective is to provide a computationally efficient and robust solution that overcomes the restrictive assumptions of Proportional-Multiple Integral Observers (PMIO) and the complexity of Fuzzy Unknown Input Observers (FUIO) while ensuring real-time feasibility for embedded automotive systems. Comparative simulations and Processor-in-the-Loop (PIL) validation demonstrated that the proposed observer achieved superior accuracy, faster convergence, and lower computational cost than Full-Order Observer (FO), PMIO, and FUIO, confirming its novelty and practical potential for integration into advanced driver assistance systems to enhance safety and reduce accident risks.

## Introduction

Vehicle safety is one of the most critical requirements of modern transportation systems. A significant reduction in road accidents can be achieved if the driver and control systems have reliable access to the internal states of the vehicle during operation^1,2^. Advanced control technologies, such as Electronic Stability Control (ESC), rely on accurate knowledge of yaw velocity and side-slip angle to maintain vehicle stability and prevent dangerous scenarios such as drifting or side-slipping^3,4^. These variables are essential indicators of vehicle behavior, particularly during cornering maneuvers^5,6^. However, the direct measurement of such variables typically requires expensive and sometimes inaccurate sensors, making this solution impractical for large-scale deployment.

To overcome these limitations, observers are employed to estimate the unmeasured state variables based on the available signals^7^. However, the design of observers for vehicle systems is nontrivial, given that vehicle dynamics are inherently nonlinear. For example, the use of low-cost measurement devices such as vision systems for lateral displacement introduces strong nonlinearities, which classical linear observers cannot effectively handle^8^. To addezss with such nonlinearities, the Takagi-Sugeno (T-S) fuzzy modeling framework^9^ has been widely adopted. This framework approximates nonlinear systems as a convex combination of Linear Time-Invariant (LTI) subsystems weighted by activation functions, enabling rigorous analysis and controller or observer design using linear system tools. Several studies have confirmed its applicability to vehicle systems^10–15^.

In recent years, the T-S fuzzy framework has been leveraged to design advanced observers and controllers for vehicle dynamics. For example^16^, developed a T-S fuzzy unknown input observer capable of estimating the lateral speed, steering input, and engine torque, which was validated in real-time hardware-in-the-loop (HIL) experiments. Similarly^11^, proposed a robust $H_\infty$control strategy based on T-S fuzzy modeling for direct yaw moment control, significantly improving handling stability. Other researchers have combined the T-S fuzzy approach with artificial intelligence methods. For instance^17^, integrated dynamic observers with neural networks for simultaneous state estimation and sensor fault diagnosis in autonomous vehicles, whereas^18^designed a cascade T-S fuzzy observer using sliding mode techniques for systems with uncertainties and unknown inputs. More recently^19^, introduced a neural network-enhanced T-S fuzzy observer for lateral speed estimation under extreme driving conditions, which was validated through experimental testing.

While these works show the potential of T-S fuzzy observers, they also reveal persistent challenges. The Proportional–Multiple Integral Observer (PMIO) proposed in^20^ was designed to estimate both the unmeasured states and unknown inputs while attenuating disturbances. However, its design requires iterative Linear Matrix Inequality (LMI)-based parameter identification, which is highly sensitive to initialization and assumes that higher-order derivatives of unknown inputs vanish, which rarely holds in practice. Similarly, the Fuzzy Unknown Input Observer (FUIO) presented in^21^ for implicit T-S models with unmeasurable premise variables performs simultaneous estimation of states and unknown inputs in a closed-loop setting. While elegant, this structure increases the algorithmic complexity, introduces additional tuning parameters, and makes the observer highly sensitive to variations in the unknown input, which can even lead to instability.

From the above review, it is evident that the existing observer designs have notable limitations. Some approaches, such as PMIO, rely on restrictive assumptions that limit their applicability, while others, such FUIO, introduce significant algorithmic and computational complexity. Moreover, many of these methods have been confined to simulation-based studies and lack validation in real-time embedded environments, which are crucial for automotive applications. These shortcomings highlight the need for a new observer design that is both computationally efficient and practically feasible. Such an observer should guarantee robust state estimation, effectively decouple the influence of unknown inputs, and be readily deployable within the embedded vehicle systems.

To address these challenges, this study proposes a novel T-S functional observer for vehicle systems. The contributions of this study are as follows.

- Robust observer design: A computationally efficient observer was developed using the T-S fuzzy framework. By decoupling the estimation of unknown inputs from the reconstruction of the state variables, the proposed design guarantees accurate state estimation independent of unknown input dynamics. Once the state variables are reconstructed, unknown inputs can be obtained under standard observability conditions without degrading stability.
- $H_\infty$-based robustness: The observer parameters were computed by solving the LMI conditions with an $H_\infty$ performance index, ensuring robustness against external disturbances and modeling uncertainties.
- Comparative evaluation: The proposed observer is systematically compared with existing approaches, namely PMIO, FUIO, and conventional Full-Order Observer (FO). The results highlight the superiority of the proposed design in terms of its robustness, accuracy, and computational efficiency.
- Real-time validation: In addition to simulation studies, the observer was validated using Processor-in-the-Loop (PIL) experiments, demonstrating its feasibility for real-time implementation in embedded automotive platforms.

The remainder of this paper is organized as follows. Sect. "Vehicle model description" presents the T-S fuzzy vehicle model, describing both measured and unmeasured states as well as known and unknown inputs. Sect. “Observer design” details the design of the proposed T-S functional observer and the associated LMI-based conditions. Sect. “Simulation results” reports the comparative simulation results for the PMIO, FUIO, and FO observers, followed by PIL-based validation. Finally, Sect. “Conclusion” summarizes the findings and outlines future research directions.

### Objectives of the research

The objectives of this study are as follows:

- To design a computationally efficient T-S fuzzy functional observer that decouples state estimation from unknown input estimation.
- To ensure robustness against disturbances and modeling uncertainties using an $H_\infty$-based LMI formulation.
- To evaluate the proposed observer against baseline methods (FO, PMIO, FUIO) through comparative simulations.
- To validate real-time feasibility using Processor-in-the-Loop (PIL) experiments.

## Vehicle model description

**Fig. 1:**
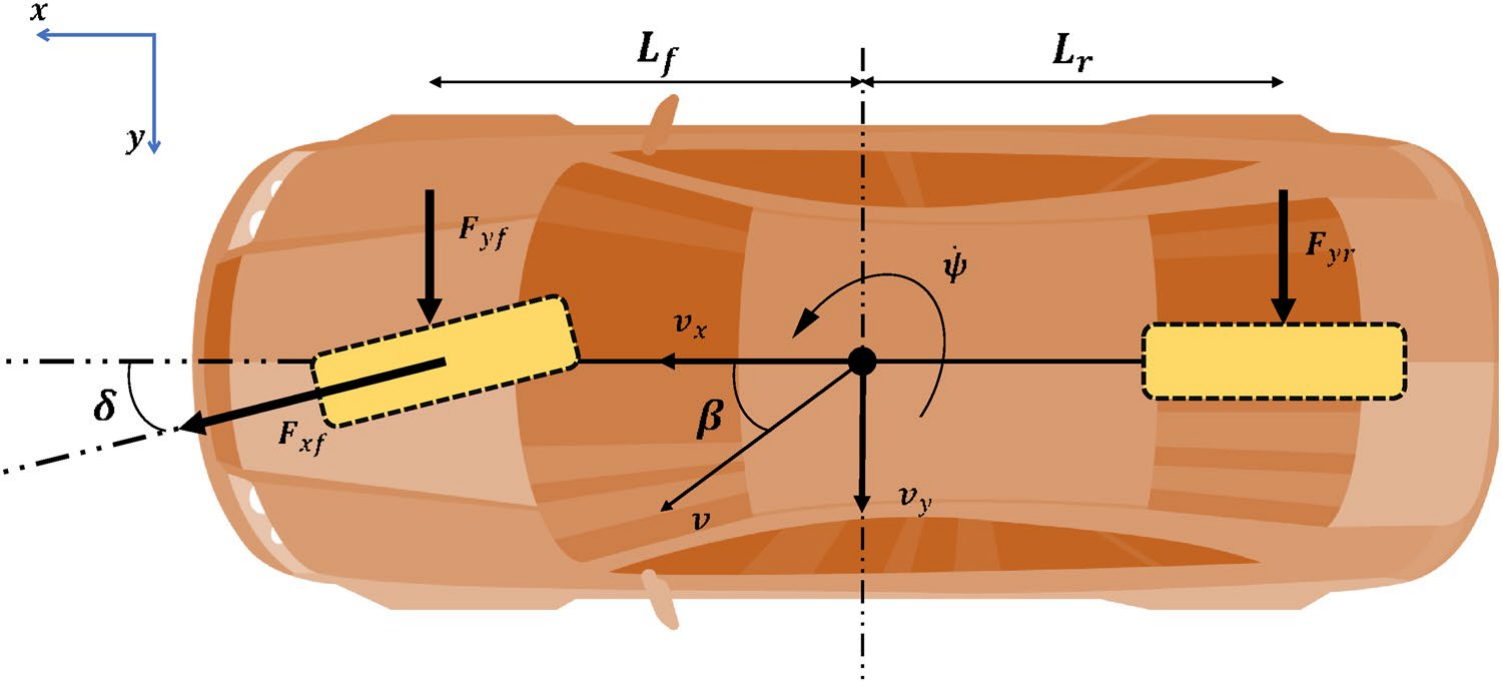
Bicycle model with steering angle control on the wheel.

In this section, the bicycle model is employed in the context of automotive dynamics as a simplified representation to estimat the transverse and longitudinal behavior of a vehicle. The vehicle is modeled with only the front and rear axles^6,22^. This formulation is also widely used for estimating the lateral interaction forces between the tires and the road surface^23–25^, as well as for capturing variables related to the vehicle’s lateral behavior^26^. The bicycle model assumes a planar, symmetrical vehicle simplified by two virtual wheels positioned at the center of each axle, as shown in Fig. 1.

Considering the bicycle model shown in Fig. 1, the following simplifying assumptions were made:

- Steering angles are small,
- Pitch, roll, and bounce motions are negligible,
- No relative yaw exists between the vehicle and the road,
- Road curvature is constant during turns and zero on straight lanes,
- Vehicle speed remains constant.

These assumptions facilitate the modeling process and observer design; however, they also constrain the applicability of the approach in real-world scenarios. Deviations from these conditions—such as large steering angles, variable road curvature, or time-varying vehicle speed—introduce nonlinearities that may lead to modeling errors. In such cases, the model must be reformulated, which can reduce estimation accuracy and subsequently degrade the observer’s performance. Therefore, although the bicycle model is suitable for moderate driving conditions, robust estimation techniques are required to handle realistic variations in vehicle motion and road geometry.

The bicycle model is controlled by the steering angle of the virtual front wheel, denoted by $\delta (t)$, which governs the yaw rate $\dot{\psi }(t)$ and direction of the vehicle velocity expressed through the sideslip angle $\beta (t)$. Based on the fundamental laws of dynamics, and considering the front $F_{yf}(t)$ and rear $F_{yr}(t)$ lateral forces, the following differential equations were obtained. Assuming that the magnitude of the vehicle’s center of gravity velocity $V_{G}(t)$ remains constant, the system can be expressed as^27,28^:

1 $$\begin{aligned} \left\{ \begin{array}{l} m v(t)\big (\dot{\beta }(t)+\dot{\psi }(t)\big )=2 F_{yf}(t)+2 F_{yr}(t), \\[6pt] J_{zz} \ddot{\psi }(t)=2 F_{yf}(t) L_{f}-2 F_{yr}(t) L_{r}, \end{array} \right. \end{aligned}$$

where *m* is the vehicle mass, $J_{zz}$ is the yaw inertia, and $L_{f}$ and $L_{r}$ denote the distances from the center of gravity to the front and rear axles, respectively.

Using nonlinear functions, the forces $F_{yf}(t)$ and $F_{yr}(t)$ can be modeled using the Pacejka model^27^, which has been employed in several studies. Based on this model, the front and rear lateral forces can be expressed as follows:

2 $$\begin{aligned} \left\{ \begin{array}{l} F_{y f}(t)=D_{f} \sin \left[ C_{f} \tan ^{-1} \left( B_{f}\left( 1-E_{f}\right) \alpha _{f}(t)+E_{f} \tan ^{-1}\left( B_{f} \alpha _{f}(t)\right) \right) \right] \\[0.8em] F_{y r}(t)=D_{r} \sin \left[ C_{r} \tan ^{-1} \left( B_{r}\left( 1-E_{r}\right) \alpha _{r}(t)+E_{r} \tan ^{-1}\left( B_{r} \alpha _{r}(t)\right) \right) \right] \end{array}\right. \end{aligned}$$

where

$$\begin{aligned} \alpha _{f}(t)=\delta (t)-\frac{L_{f} \dot{\psi }(t)}{v(t)}-\beta (t), \qquad \alpha _{r}(t)=\frac{L_{r} \dot{\psi }(t)}{v(t)}-\beta (t). \end{aligned}$$

The coefficients $B_{i}$, $C_{i}$, $D_{i}$, and $E_{i}$ with $(i=f, r)$ are related to the tire characteristics, as well as other factors, such as the vehicle’s operating conditions and road adhesion coefficient. Angles $\alpha _{f}(t)$ and $\alpha _{r}(t)$ represent the slip angles of the front and rear tires, respectively.

In other studies^29,30^, the lateral forces $F_{yi}(t)$ with $(i=f, r)$ are assumed to be proportional to the slip angles $\alpha _{i}(t)$, and are represented by the linear relations $F_{yf}(t)=C_{f} \alpha _{f}(t)$ and $F_{yr}(t)=C_{r} \alpha _{r}(t)$.

**Fig. 2:**
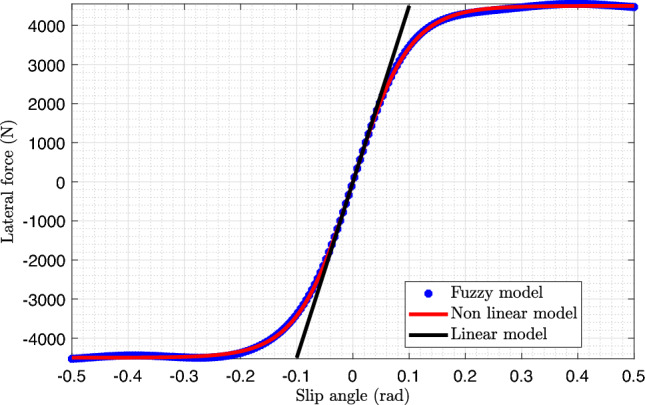
Front lateral force as a function of slip angles (dotted line: fuzzy model; solid red line: nonlinear model; solid black line: linear model)^31^.

Figure 2 illustrates the variation in the lateral forces as a function of the slip angles. It can be observed that the linear approximation of the lateral forces is only valid for slip angles close to zero (i.e., small values of $\alpha _{i}(t)$). For larger slip angles, the relationship becomes nonlinear, thereby necessitating a more sophisticated modeling approach.

In this work, the nonlinear characteristics were captured through a T-S fuzzy modeling framework. Specifically, two fuzzy rules were considered to distinguish between small and large slip angles, represented by fuzzy sets $M_{1}$ and $M_{2}$. These rules govern both the front and rear lateral force models.

3 $$\begin{aligned} \begin{aligned}&\text { If }\left| \alpha _{f}(t)\right| \text { is } M_{1} \text { then }\left\{ \begin{array}{l} F_{y f}(t)=C_{f 1} \alpha _{f}(t) \\ F_{y r}(t)=C_{r 1} \alpha _{r}(t) \end{array}\right. \\&\text { If }\left| \alpha _{f}(t)\right| \text { is } M_{2} \text { then }\left\{ \begin{array}{l} F_{y f}(t)=C_{f 2} \alpha _{f}(t) \\ F_{y r}(t)=C_{r 2} \alpha _{r}(t) \end{array} .\right. \end{aligned} \end{aligned}$$

It should be noted that $C_{f j}$ and $C_{r j}$ $(j=1,2)$ represent the cornering stiffnesses of the front and rear tires, respectively.

To identify the overall forces^32^, we have

4 $$\begin{aligned} \left\{ \begin{array}{l} F_{y f}(t)=h_{1}\left( \left| \alpha _{f}(t)\right| \right) C_{f 1} \alpha _{f}(t)+h_{2}\left( \left| \alpha _{f}(t)\right| \right) C_{f 2} \alpha _{f}(t) \\ F_{y r}(t)=h_{1}\left( \left| \alpha _{f}(t)\right| \right) C_{r 1} \alpha _{r}(t)+h_{2}\left( \left| \alpha _{f}(t)\right| \right) C_{r 2} \alpha _{r}(t) \end{array}\right. \end{aligned}$$

The membership function of $M_{i}$ is expressed by the variable $h_{i}$ $(i=1,2)$. These satisfy the following conditions:

5 $$\begin{aligned} \left\{ \begin{array}{l} \sum _{i=1}^{2} h_{i}\left( \left| \alpha _{f}(t)\right| \right) =1 \\ 0 \le h_{i}\left( \left| \alpha _{f}(t)\right| \right) \le 1, \quad i=1,2 \end{array}\right. \end{aligned}$$

It is noted that the membership function $h_{i}(t)$ is expressed as follows:

6 $$\begin{aligned} h_{i}\left( \left| \alpha _{f}(t)\right| \right) =\frac{\omega _{i}\left( \left| \alpha _{f}(t)\right| \right) }{\sum _{i=1}^{2} \omega _{i}\left( \left| \alpha _{f}(t)\right| \right) } , \quad i=1,2 \end{aligned}$$

where,

$$\omega _{i}\big (\big |\alpha _{f}(t)\big |\big )=\frac{1}{\bigg (1+\bigg |\,\bigg (\frac{|\alpha _{f}(t)| -c_{i}}{a_{i}}\bigg )\bigg |\bigg )^{2 b_{i}}}, \quad i=1,2$$

The Levenberg-Marquardt algorithm^33^, in combination with the least squares method, forms the basis of one of the identification approaches for determining the parameters of the membership functions $\big (a_{i}, b_{i}, c_{i}\big )$, as well as the stiffness coefficients $C_{f j}$ and $C_{r j}$ $(j = 1, 2)$. These variables assume the following values for $\mu = 0.7$:

$$\begin{aligned} a_{1}= & 0.0978, \quad b_{1}=0.7079, \quad c_{1}=0.0137, \quad C_{f 1}=69120, \quad C_{r 1}=56458 \\ a_{2}= & 0.1924, \quad b_{2}=0.7445, \quad c_{2}=-1.1388, \quad C_{f 2}=-796.64, \quad \text {and} \quad C_{r 2}=-876.24. \end{aligned}$$

Considering the equation of model (1) and substituting the nonlinear lateral forces with their corresponding values according to the T-S fuzzy rules, model (1) can be reformulated as follows:

7 $$\begin{aligned} \begin{aligned} \begin{bmatrix} \dot{\beta }(t) \\ \ddot{\psi }(t) \end{bmatrix}&= \sum _{i=1}^{2} h_{i}\left( \left| \alpha _{f}(t)\right| \right) \begin{bmatrix} a_{11 i} & a_{12 i} \\ a_{21 i} & a_{22 i} \end{bmatrix} \begin{bmatrix} \beta (t) \\ \dot{\psi }(t) \end{bmatrix} + \sum _{i=1}^{2} h_{i}\left( \left| \alpha _{f}(t)\right| \right) \begin{bmatrix} b_{1 i} \\ b_{2 i} \end{bmatrix} \delta (t) \end{aligned} \end{aligned}$$

where,

$$\begin{aligned} a_{11 i}= & -2 \frac{C_{r i}+C_{f i}}{m v}, \qquad a_{12 i}=-1-2 \frac{L_{f} C_{f i}-L_{r} C_{r i}}{m v^{2}} \\ a_{21 i}= & -2 \frac{L_{f} C_{f i}-L_{r} C_{r i}}{J_{z z}}, \qquad a_{22 i}=-2 \frac{L_{f}^{2} C_{f i}+L_{r}^{2} C_{r i}}{J_{z z} v} \\ b_{1 i}= & 2 \frac{C_{f i}}{m v}, \qquad \text {and} \qquad b_{2 i}=2 \frac{L_{f} C_{f i}}{J_{z z}} . \end{aligned}$$

**Fig. 3:**
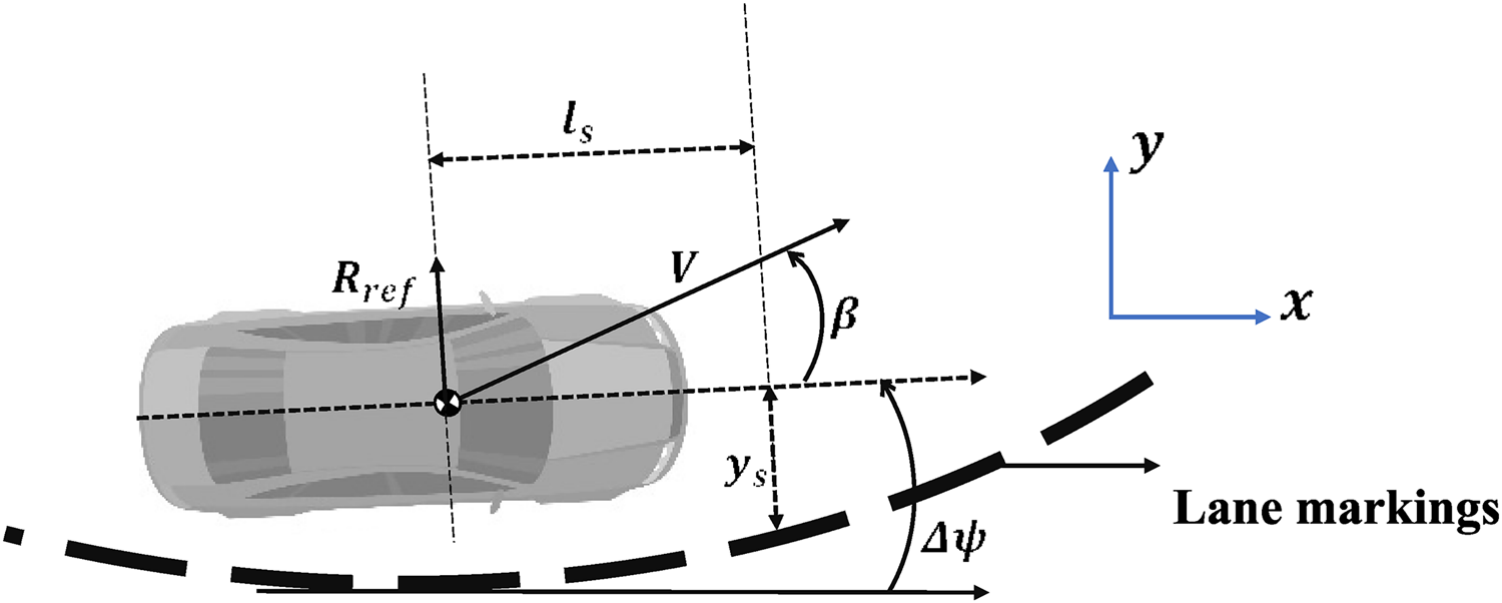
Vision system of the vehicle.

During the driving experiment, a vision system was employed to measure the lateral displacement of the vehicle from its front, as shown in Fig. 3. This measurable state, denoted by $y_{s}(t)$, represents the lateral position throughout the experiment. It is influenced by the road geometry as well as the vehicle’s motion along the road, and can be expressed by the following equation:

8 $$\begin{aligned} \dot{y_{s}}(t)=v(t)(\beta (t)+\Delta \psi (t))+l_{s} \Delta \dot{\psi }(t) \end{aligned}$$

From Eq. (8), *v*(*t*) denotes the vehicle speed during motion, $l_s$ represents the distance from which the measurement is obtained, and $\Delta \psi (t)$ corresponds to the angular displacement, defined as the angle between the road tangent and the axis passing through the vehicle’s center of gravity along the direction of travel. The angular displacement $\Delta \psi (t)$ is expressed as follows:

9 $$\begin{aligned} \Delta \dot{\psi }(t) =\dot{\psi }(t)-\frac{v(t)}{R_{c}(t)}=\dot{\psi }(t)- v(t)w(t) \end{aligned}$$

where road curvature *w*(*t*) acts as an important unknown input to the system.

By combining Eq. (7), which governs the lateral and yaw dynamics of the vehicle, using Eq. (8), describing the evolution of the measured lateral displacement $y_s(t)$, and Eq. (9), which relates the angular displacement $\Delta \psi (t)$ to the yaw rate and road curvature, the dynamics of $y_s(t)$ can be rewritten by substituting (9) into (8) as

$$\dot{y_s}(t) = v(t) \big (\beta (t) + \Delta \psi (t)\big ) + l_s \big (\dot{\psi }(t) - v(t) w(t)\big ).$$

Defining the augmented state vector

$$x(t) = \begin{bmatrix}\beta (t)&\dot{\psi }(t)&y_s(t)&\Delta \psi (t)\end{bmatrix}^T,$$

the system dynamics can then be expressed in the extended T–S form:

10 $$\begin{aligned} \left\{ \begin{array}{lcl} \dot{x}(t) & = & \sum _{i=1}^{2} h_{i}\left( \left| \alpha _{f}(t)\right| \right) \begin{bmatrix} a_{11 i} & a_{12 i} & 0 & 0 \\ a_{21 i} & a_{22 i} & 0 & 0 \\ v & l_{s} & 0 & v \\ 0 & 1 & 0 & 0 \end{bmatrix} \begin{bmatrix} \beta (t) \\ \dot{\psi }(t) \\ y_{s}(t) \\ \Delta \psi (t) \end{bmatrix} \\ & & + \sum _{i=1}^{2} h_{i}\left( \left| \alpha _{f}(t)\right| \right) \begin{bmatrix} b_{1 i} \\ b_{2 i} \\ 0 \\ 0 \end{bmatrix} \delta (t) + \begin{bmatrix} 0 \\ 0 \\ -l_{s} v \\ -v \end{bmatrix} w(t) \\ y(t) & = & Cx(t) \\ \end{array} \right. \end{aligned}$$

It is considered that $\delta (t)$ is a known input, whereas *w*(*t*) is an unknown input. Because $y_s(t)$ is treated as a measured variable, the output matrix is defined as

$$C = \begin{bmatrix} 0&0&1&0 \end{bmatrix}.$$

Accordingly, model (10) can be further simplified into the following form:

11 $$\begin{aligned} (\Sigma ): \left\{ \begin{array}{l} \dot{x}(t)=\sum _{i=1}^{2} h_{i}\left( \left| \alpha _{f}(t)\right| \right) \left[ A_{i} x(t)+B_{i} \delta (t)\right] +L w(t) \\ y(t)=C x(t) \end{array}\right. \end{aligned}$$

where,

$$\begin{aligned}&A_{i}=\left[ \begin{array}{llll} a_{11 i} & a_{12 i} & 0 & 0 \\ a_{21 i} & a_{22 i} & 0 & 0 \\ v & l_{s} & 0 & v \\ 0 & 1 & 0 & 0 \end{array}\right] , \quad B_{i}=\left[ \begin{array}{l} b_{1 i} \\ b_{2 i} \\ 0 \\ 0 \end{array}\right] , \quad \text {and} \quad L=\left[ \begin{array}{l} 0 \\ 0 \\ -l_{s} v \\ -v \end{array}\right] . \end{aligned}$$

The system state matrix is denoted as $A_i \in \mathbb {R}^{4 \times 4}$, and $B_i \in \mathbb {R}^{4 \times 1}$ represents the control input matrix associated with the known inputs of the system. The matrix $L \in \mathbb {R}^{4 \times 1}$ characterizes the influence of an unknown input. On the other hand, the observation matrix is given by $C \in \mathbb {R}^{1 \times 4}$.

## Observer design

First, vector $z(t) = F x(t)$ is defined as the quantity to be estimated, where $F \in \mathbb {R}^{3 \times 4}$ is a constant matrix with rank $rank(F) = 3$. Moreover, the matrix $$\begin{bmatrix} C \quad F \end{bmatrix}^T$$ must be nonsingular to ensure that the numerical value of *F* can be determined in the presence of unmeasured system states. Consequently, this vector can be expressed as:

$$F = \begin{bmatrix} 1 & 0 & 0 & 0 \\ 0 & 1 & 0 & 0 \\ 0 & 0 & 0 & 1 \end{bmatrix}$$

Thus, in this case, the vector *z*(*t*) is given by

12 $$\begin{aligned} z(t) = \begin{bmatrix} \beta (t) \quad \dot{\psi }(t) \quad \Delta \psi (t) \end{bmatrix}^T \in \mathbb {R}^{3 \times 1} \end{aligned}$$

In the present context, not all the states in *x*(*t*) are directly measurable. To address this, a T-S observer is designed to estimate the unknown states and inputs of the system. The dynamics of the proposed T-S observer are described as follows:

13 $$\begin{aligned} (Obs): \left\{ \begin{array}{lcl} \dot{\bar{w}}(t) & = & \sum _{i=1}^{2} h_{i}\left( \left| \alpha _{f}(t)\right| \right) ( N_i \bar{w}(t) + J_i y(t) + H_i \delta (t)) \\ \hat{z}(t) & = & \bar{w}(t) + E y(t) \\ \end{array} \right. \end{aligned}$$

In this framework, the state vector of the proposed observer is denoted by $\bar{w}(t) \in \mathbb {R}^{3}$, and the estimated value of *z*(*t*) is represented by $\hat{z}(t) \in \mathbb {R}^{3}$. It is necessary to determine matrices $N_i \in \mathbb {R}^{3 \times 3}$, $J_i \in \mathbb {R}^{3 \times 1}$, $H_i \in \mathbb {R}^{3 \times 1}$, and $E \in \mathbb {R}^{3 \times 1}$ such that the observer estimation error converges asymptotically to zero.

**Fig. 4:**
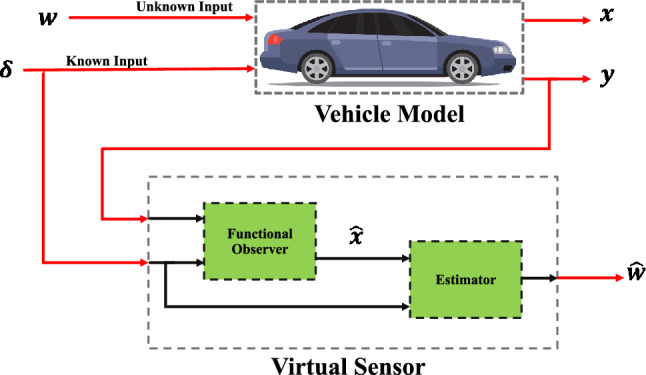
Unknown input state observer structure.

As illustrated in Fig. 4, the proposed unknown input observer can be used to estimate both *z*(*t*) and the unknown input *w*(*t*) from the available measurements, namely the input $\delta (t)$ and output *y*(*t*).

### Theorem 1

For any initial condition $\bar{w}(0)$, any known input *u*(*t*), and any unknown input *d*(*t*), the estimation error of the state converges asymptotically to zero if and only if the matrix variables $N_i$, $J_i$, $H_i$, and *E* are chosen, such that the following conditions are satisfied:

1. $N_i$ is a Hurwitz matrix.
2. $$P A_i - N_i P - J_i C = 0$$.
3. $$P L = 0$$.
4. $$H_i = P B_i$$.

Here, matrix *P* is defined as $P = F - E C$.

### Proof

The following equation represents the dynamics of the observation error.

14 $$\begin{aligned} e(t) = z(t) - \hat{z}(t) = F x(t) - \hat{z}(t) = Px(t) - \bar{w}(t) \end{aligned}$$

By differentiating *e*(*t*) and substituting the expressions for $\dot{x}(t)$ and $\dot{\bar{w}}(t)$, the error dynamics can be written as:

15 $$\begin{aligned} \dot{e}(t)&= \sum _{i=1}^{2} h_{i}\left( \left| \alpha _{f}(t)\right| \right) (N_i e(t) + (P A_i - N_i P - J_i C) x(t) \nonumber \\&\quad + (H_i - P B_i) \delta (t) + PL w(t)) \end{aligned}$$

Equation (15) can be simplified to the following form by applying the conditions for determining the matrices $N_i$, $J_i$, $H_i$, and *E*, corresponding to requirements 1), 2), 3), and 4):

16 $$\begin{aligned} \dot{e}(t) = \sum _{i=1}^{2} h_{i}\left( \left| \alpha _{f}(t)\right| \right) N_i e(t) \end{aligned}$$

In the following, the design of the proposed T-S functional observer (13) for predicting the value of *z*(*t*), based on the observer system (11), primarily relies on determining the matrices $N_i$, $J_i$, $H_i$, and *E* while satisfying the previously stated requirements 1), 2), 3), and 4). $\square$

The following equation defines a full-row rank matrix, where $T_1 \in \mathbb {R}^{1 \times 3}$:

17 $$\begin{aligned} \left. \left[ \begin{array}{c} F \\ T_1 \end{array}\right] = \left[ \begin{array}{cc} H_{1}&E_{1} \end{array}\right] ^{-1} \right. \end{aligned}$$

Using the expression of *P* and requirement 2) from Theorem 1, matrix $N_i$ can be expressed as

18 $$\begin{aligned} \left. \begin{array}{cc} N_i = FA_iH_{1}-[\begin{array}{cc} E & K_i \end{array}]\left[ \begin{array}{c} CA_i \\ C \end{array}\right] H_{1} \end{array}\right. \end{aligned}$$

where,

19 $$\begin{aligned} \left. K_i = J_i -N_i E \right. \end{aligned}$$

Consequently, according to conditions 3) and 4) from Theorem 1, the next requirement is satisfied by the unidentified matrix $$[\begin{array}{cc} E&K_i \end{array}]$$.

20 $$\begin{aligned} \left. [\begin{array}{cc}E&K_i\end{array}] M_{1i} = M_{2i} \right. \end{aligned}$$

Note that the matrices $M_{1i}$ and $M_{2i}$ are written in the form:

21 $$\begin{aligned} \left\{ \begin{array}{lcl} M_{1i} = \left[ \begin{array}{ccccc} CA_iE_{1} & CL \\ CE_{1} & 0 \end{array}\right] \\ M_{2i} = \left[ \begin{array}{ccccc} FA_iE_{1} & FL \\ \end{array}\right] \end{array} \right. \end{aligned}$$

According to^34^, the necessary and sufficient condition for the solution of (20) is

22 $$\begin{aligned} rank\left[ \begin{array}{ccccc} FA_i & FL \\ CA_i & CL \\ C & 0 \\ F & 0 \end{array} \right] = rank\left[ \begin{array}{ccccc} CA_i & CL\\ C & 0 \\ F & 0 \end{array}\right] \end{aligned}$$

Under condition (22), the general solution to Eq. (20) can be expressed as

23 $$\begin{aligned} \left. [\begin{array}{cc} E&Ki \end{array}]\right. = M_{2i}M_{1i}^{+} + Z(I - M_{1i}M_{1i}^{+}) \end{aligned}$$

Here, *Z* represents an arbitrary matrix with the appropriate dimensions.

Substituting the solution from (23) into (18) yields a new form of the matrix $N_i$, expressed as follows.

24 $$\begin{aligned} N_i = N_{1i} - ZN_{2i} \end{aligned}$$

where,

25 $$\begin{aligned} \left\{ \begin{array}{lcl} N_{1i} = F A_i H_{1} - M_{2i}M_{1i}^{+}\left[ \begin{array}{c} CA_i \\ C \end{array}\right] H_{1}\\ N_{2i} = (I - M_{1i}M_{1i}^{+})\left[ \begin{array}{c} CA_i \\ C \end{array}\right] H_{1}\ \end{array} \right. \end{aligned}$$

To ensure the asymptotic stability of the observation error, the matrix $N_i$ must be appropriately selected. According to Lyapunov’s theorem, the observer is guaranteed to converge when the Lyapunov function is considered:

$$\forall e(t) \ne 0 \left\{ \begin{array}{lcl} V(e(t))> 0 \\ \dot{V}(e(t)) < 0 \end{array} \right.$$

Using Eq.(16), that gives us an inequality in the form:

26 $$\begin{aligned} \forall e(t) \ne 0 \left\{ \begin{array}{lcl} X> 0 \\ N_i^{T}X + XN_i < 0 \end{array} \right. \end{aligned}$$

Applying the Schur complement^35,36^, another way to write Eq. (26) is as follows:

$$\begin{pmatrix} -X & 0\\ 0 & N_i^{T}X + XN_i \end{pmatrix} < 0$$

Equation (24) allows us to formulate the following LMI:

27 $$\begin{aligned} \begin{pmatrix} -X & 0\\ 0 & N_{1i}^{T}X - N_{2i}^{T} Q^{T} + X N_{1i} - Q N_{2i} \end{pmatrix} < 0 \end{aligned}$$

where $$Z = X^{-1}Q$$.

To calculate the value of $J_i$, we start with Eq. (19), we obtain

28 $$\begin{aligned} \left. J_i = K_i + N_i E \right. \end{aligned}$$

According to requirement 4) of Theorem 1, the following expression holds:

29 $$\begin{aligned} \left. H_i = PB_i \right. \end{aligned}$$

To ensure that the estimation error $e(t) = z(t) - \hat{z}(t)$ asymptotically approaches zero, parameter $N_i$ can be determined using Eq. (24) and the expression of *Z*, which is obtained by solving the LMI in (27).

The following expression can then be used to estimate the states of the actual system $\hat{x}(t)$:

30 $$\begin{aligned} \hat{x}(t) = \begin{bmatrix} C \\ F \end{bmatrix}^{-1} \begin{bmatrix} y(t) \\ \hat{z}(t) \end{bmatrix} \end{aligned}$$

The actual value of the unknown input *w*(*t*) can be estimated using the following formula:

31 $$\begin{aligned} \hat{w}(t) = L^{+}\sum _{i=1}^{2} h_{i}\left( \left| \alpha _{f}\right| \right) (\dot{\hat{x}}(t) - A_i\hat{x}(t) - B_i\delta (t)) \end{aligned}$$

## Simulation results

In this section, the results obtained after applying the suggested T-S observer (13) to the T-S fuzzy model (11) are presented. Mathematical calculations were performed using MATLAB, and simulations were conducted in Simulink. Table 1 presents the parameters of a car traveling at a constant speed of $20 \,[\text {m/s}]$.

**Table 1:** Vehicle parameters’ nominal values.

*m* (kg)	$J_{zz}$ (kg$\cdot$m$^2$)	$L_f$ (m)	$L_r$ (m)	*v* (m/s)
1500	2454	1.0065	1.4625	20

**Fig. 5:**
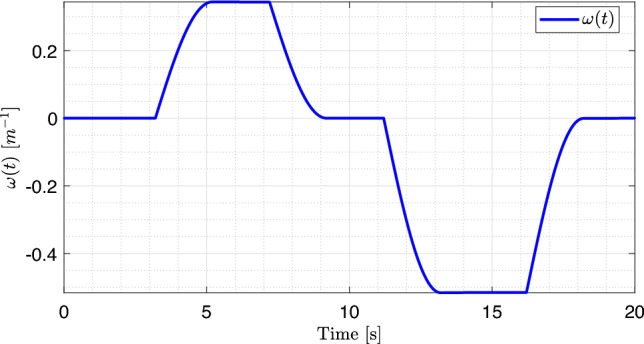
Road curvature *w*(*t*).

**Fig. 6:**
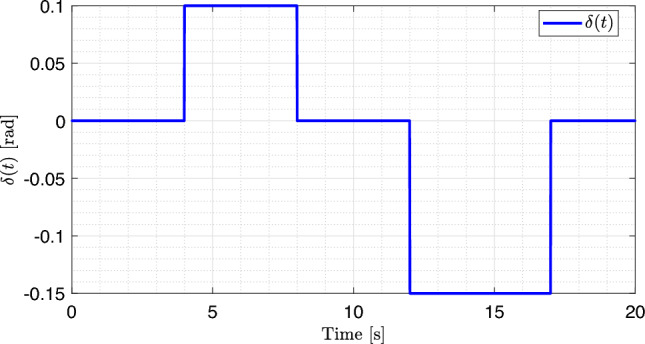
Steering angle $$\delta (t)$$.

For the known and unknown inputs of the system, namely the steering angle $\delta (t)$ and road curvature *w*(*t*), two signals were applied to represent the variations in $\delta (t)$ and *w*(*t*) over time during the experiment, as illustrated in Figs. 5 and 6. The road curvature is treated as a reference input because its values are required for comparison with the estimator’s output, as shown in Fig. 4.

Road curvature as a function of time is shown in Fig. 5. The curvature initially assumes a value of zero and then increases sinusoidally to a positive value before returning to zero. Subsequently, it decreases to a negative value in a sinusoidal manner and returns once again to zero. The steering angle, as a function of time, is shown in Fig. 6. The steering angle exhibits a staircase profile, changing its value at specific time instants: it begins at zero, increases to a constant positive value, returns to zero, decreases to a constant negative value, and finally returns to zero. Owing to the direct relationship between the steering angle of the vehicle and road curvature, the two signals are strongly correlated.

To determine the values of the functional observer parameters, the LMI problem in (27) is solved using the YALMIP toolbox^37^. Accordingly, in accordance with (24), (23), (28), and (29), the resulting parameters $N_i$, *E*, $J_i$, and $H_i$ are obtained as follows:

$$\begin{aligned} N_1= & \begin{bmatrix} -8.0534 & -0.9108 & 0 \\ 21.8186 & -7.7436 & 0 \\ -2.0000 & 0 & -2.0000 \end{bmatrix} \text {,} \quad N_2 = \begin{bmatrix} -5.4927 & -0.9945 & 0 \\ 1.3347 & -4.9725 & 0 \\ -2.0000 & 0 & -2.0000 \end{bmatrix}\text {,} \\ J_1= & \begin{bmatrix} 0 \\ 0 \\ -0.2000 \end{bmatrix}\text {,} \quad J_2 = \begin{bmatrix} 0 \\ 0 \\ -0.2000 \end{bmatrix}\text {,} \quad H_1 = \begin{bmatrix} 4.0475 \\ 49.8022 \\ 0 \end{bmatrix} \text {,} \\ H_2= & \begin{bmatrix} 3.2093 \\ 39.4889 \\ 0 \end{bmatrix} \text {,} \quad \text {and} \quad E = \begin{bmatrix} 0 \\ 0 \\ 0.1000 \end{bmatrix} \text {.} \end{aligned}$$

For comparison, three additional observers were included in the simulation. First, as given in Eq. 32, is a well-known full-order observer (FO) that reconstructs all unmeasured states of a dynamic system from available inputs and outputs^38^, providing accurate state estimation for control and monitoring applications. Second, FUIO, shown in Eq. 33 and proposed by^21^, simultaneously estimates both unmeasurable states and unknown inputs of continuous-time T-S implicit systems with unmeasurable premise variables, ensuring accurate and exponentially convergent reconstruction via an augmented system structure, singular value decomposition, and Lyapunov-based stability conditions formulated as LMIs. Finally, the PMIO, as represented in Eq. 34 and introduced by^20^, concurrently estimates the states and unknown inputs of T-S fuzzy systems with unmeasurable premise variables, reducing conservatism using a non-quadratic Lyapunov function.

32 $$\begin{aligned} & \left\{ \begin{aligned} \dot{w}_{FO}(t)&= \sum _{i=1}^{2} h_i \left( N_{FOi} w_{FO}(t) + J_{FOi} y(t) + H_{FOi} u(t) \right) \\ \hat{x}(t)&= w_{FO}(t) - E_{FO} y(t) \end{aligned} \right. \end{aligned}$$

33 $$\begin{aligned} & \left\{ \begin{aligned} \dot{w}_{FUIO}(t)&= \sum _{i=1}^{2} h_i \left( N_{FUIOi} w_{FUIO}(t) + L_{FUIO1i} y(t) + L_{FUIO2i} y(t) + G_{FUIOi} u(t) \right) \\ \hat{z}(t)&= w_{FUIO}(t) + \Psi _2 y(t) + K_{FUIO} \Psi _4 y(t) \end{aligned} \right. \end{aligned}$$

34 $$\begin{aligned} & \left\{ \begin{aligned} \dot{\hat{x}}&= \sum _{i=1}^{2} h_i \left( A_i \hat{x}(t) + B_i u(t) + L \hat{d}(t) \right) + L_p \left( y(t) - C \hat{x}(t) \right) \\ \dot{\hat{d}}(t)&= L_{10} \left( y(t) - \hat{y}(t) \right) + \hat{d}_1(t) \\ \dot{\hat{d}}_1(t)&= L_{11} \left( y(t) - \hat{y}(t) \right) + \hat{d}_2(t) \\&\vdots \\ \dot{\hat{d}}_{q-1}(t)&= L_{1_{q-1}} \left( y(t) - \hat{y}(t) \right) + \hat{d}_q(t) \end{aligned} \right. \end{aligned}$$

A two-stage evaluation process is conducted to validate the proposed T-S observer: Model-in-the-Loop (MIL) and Processor-in-the-Loop (PIL). The MIL stage focuses on the simulation-based verification of the observer’s ability to estimate critical but unmeasured vehicle states, such as the side slip angle, yaw rate, angular displacement, and road curvature, under controlled initial conditions and parameter variations. This allows for a fair comparison with existing approaches, including FO, PMIO, and FUIO observers. The PIL stage then assesses the feasibility of real-time implementation on the embedded hardware, ensuring that the observer maintains its accuracy and stability when subject to computational constraints. Together, these stages provide a comprehensive validation of both the estimation accuracy and practical deployability.

For reproducibility, the simulation setup is described as follows: A fixed-step discrete solver was used in Simulink with a sampling time of $T_s = 0.01 s$ s and a total simulation horizon of 20 s. The implementation relies on a combination of MATLAB Function blocks and Simulink subsystems. Observers were realized through both MATLAB Function blocks and direct construction using gain and integrator blocks. Linear algebra operations were performed using the default Simulink solver in conjunction with custom MATLAB routines, where necessary. This configuration ensures consistent numerical treatment across all tested observers (FO, PMIO, FUIO, and the proposed T-S observer).

### Model-in-the-Loop (MIL) validation

An experiment was conducted to estimate the unmeasured system states and assess the performance of the proposed observer. Particular attention was given to the side-slip angle, yaw rate, angular displacement, and road curvature, as these variables are essential for accurately representing vehicle dynamics.

**Fig. 7:**
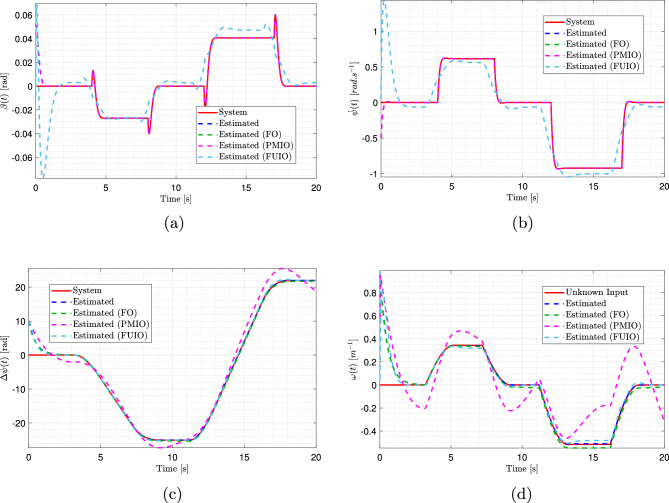
Estimation results of side slip angle, yaw rate, angular displacement, and road curvature using multiple observers. (Solid red: actual system response; dashed blue: proposed observer estimate; dashed green: FO estimate; dashed magenta: PMIO estimate; dashed light blue: FUIO estimate).

**Fig. 8:**
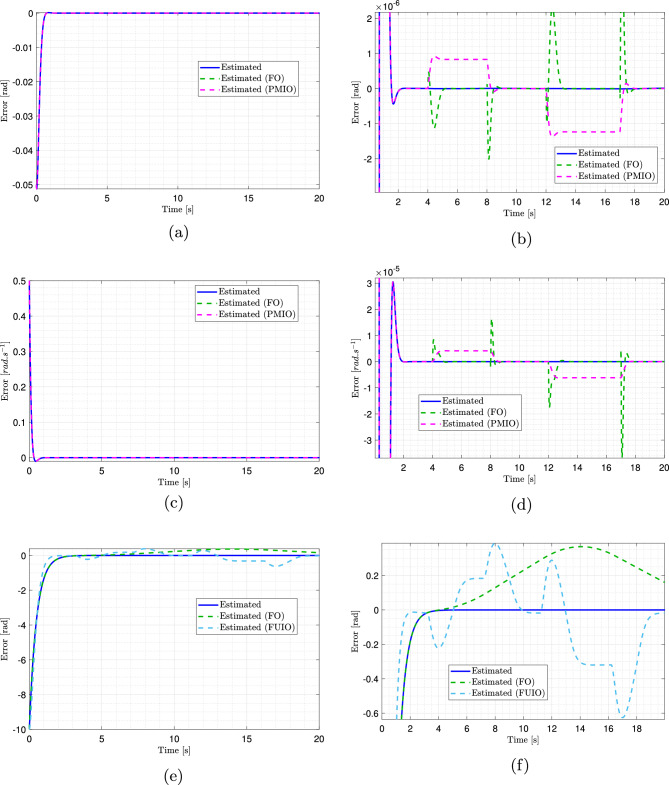
Estimation errors of side slip angle, yaw speed, and angular displacement, with zoomed views on the right. (Solid blue: proposed observer estimate; dashed green: FO estimate; dashed magenta: PMIO estimate; dashed light blue: FUIO estimate).

The unmeasured system states can now be estimated for the initial conditions $$\bar{w}(0) = \begin{bmatrix} 0.05&-0.5&10 \end{bmatrix}^T$$. Fig. 7a, b, and c present the time evolution of the side slip angle $\beta (t)$, yaw rate $\dot{\psi }(t)$, and angular displacement $\Delta \psi (t)$, respectively. The results demonstrate that the estimated states closely track the actual system behavior. This accuracy is further substantiated in Fig. 8a, c, and e, which depict the estimation errors between true unmeasured states and their corresponding estimates. In all cases, the estimation errors converged asymptotically to zero, thereby confirming the effectiveness of the proposed observer.

The proposed T-S observer demonstrated superior performance compared to the alternative approaches (FO, PMIO, and FUIO) in estimating the slip angle and yaw rate, as illustrated in Fig. 7a and b. A detailed examination of the results reveals that the T-S observer provides highly accurate and responsive estimations that closely match the trajectories of the actual system. Unlike other observers, such as FUIO, which exhibits pronounced oscillations during step transitions and significant initial overshoots.

The T-S observer outperformed the alternative approaches in terms of accuracy when estimating the angular displacement, as shown in Fig. 7c. Its response exhibited excellent fidelity and was almost perfectly superimposed on that of the actual system. By contrast, the other observers, although generally effective, presented more evident tracking errors. Toward the end of the simulation, noticeable offsets and overshoots appeared, with PMIO displaying the most significant deviation.

Because directly demonstrating the effectiveness of the proposed observer in estimating the system’s state variables is challenging, estimation errors were computed and analyzed. Fig. 8a, b, c, and d illustrate the superior performance of the proposed observer compared with the alternative approaches. At first glance, all methods appear to perform well, with error values close to zero, as shown in the overview plots 8a and c for the side slip angle and yaw rate. However, the advantage of the proposed observer becomes evident in magnified views 8b and d, where its estimation errors are the smallest, converge most rapidly to zero, and remain highly stable with minimal fluctuations. In contrast, FO and PMIO exhibited more pronounced oscillations and larger error peaks, particularly during dynamic transitions, indicating lower responsiveness and accuracy.

The estimation errors for the angular displacement are shown in Fig. 8e and f. While all three observers’ errors converge to zero, as shown in Fig. 8e, the superiority of the proposed observer is highlighted in the zoomed view 8f. FO and FUIO display larger and more variable errors, with FUIO exhibiting highly unpredictable behavior toward the end of the simulation. In contrast, the T-S observer achieved near-perfect tracking throughout the entire simulation horizon and rapidly converged to the true values. Consequently, the key advantage of the proposed observer is its ability to provide highly accurate and reliable estimates of the vehicle’s state variables under all driving conditions.

**Fig. 9:**
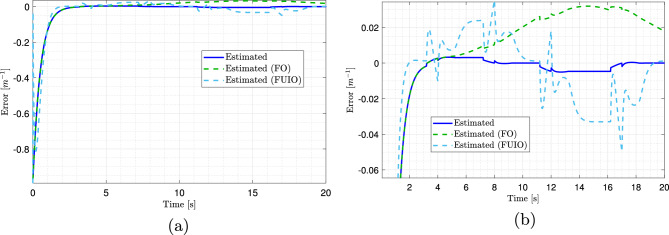
Estimation errors of the road curvature using multiple observers and their zoomed view.

Equation (31) allows the prediction of the unknown input. This is illustrated in Fig. 7d, which shows the time evolution of the road curvature, and Fig. 9a, which depicts the estimation errors between the actual unknown input and values predicted by the proposed estimator.

The road curvature estimate *w*(*t*), is shown in Fig. 7d. The curves of the proposed T-S observer are almost perfectly superimposed on the actual value of the unknown input, demonstrating their exceptional accuracy. In contrast, the other observers exhibited notable errors. The PMIO displayed significant oscillations and a large deviation from the true value, indicating that it is the least effective and unsuitable for accurate curvature estimation.

The estimation errors for the road curvature can be observed in Fig. 9a. This clearly demonstrates the rapid response of the proposed observer, which exhibits a very steep initial slope and a significantly faster convergence rate than alternative approaches. Although FO and FUIO also reach extremely low error levels, they do so gradually. The fast response and high precision of the proposed observer are further confirmed by the magnified view in Fig. 9b, which shows extremely accurate and steady tracking, stabilizing almost immediately with minimal error. In contrast, the FUIO exhibited oscillations and unpredictable behavior, whereas FO displayed larger and more variable errors. This figure further substantiates that the proposed observer outperforms the other two observes in terms of accuracy and convergence speed.

The comparative results of the four observers are summarized in Tables 2, 3, 4, and 5, which report the mean absolute errors (MAEs) for the side slip angle $\beta (t)$, yaw speed $\dot{\psi }(t)$, angular displacement $\Delta \psi (t)$, and road curvature $\omega (t)$ under variations in speed, mass, and yaw inertia. The T-S observer consistently provides the lowest MAEs across most states; for instance, the side slip angle MAE ranges from $8.68\times 10^{-4}$ to $1.57\times 10^{-2}$, yaw speed MAE ranges from $4.49\times 10^{-3}$ to $4.71\times 10^{-2}$, and road curvature MAE ranges from $2.62\times 10^{-2}$ to $1.04\times 10^{-1}$. The FO observer achieves comparable accuracy to the T-S observer for the side slip angle and yaw speed (differences within $10^{-6}$ to $10^{-5}$), but exhibits higher errors for angular displacement (up to 0.43 versus 0.25 for T-S) and road curvature (up to 0.042 versus 0.028 for T-S). The PMIO and FUOI show the largest errors across all states, e.g., angular displacement errors reach 1.80–4.67 for the PMIO and yaw speed errors up to 0.13 for the FUIO, indicating weaker overall performance. Overall, the T-S observer emerges as the most reliable approach, with the FO observer serving as a reasonable alternative for specific states where the MAEs are close to those of T-S.

**Table 2:** Average of the absolute values of side slip angle estimation error under the influence of parameter variations between 80% and 120% of the nominal values of sprung mass *m*, yaw inertia $J_{zz}$, and vehicle speed *v*.

Variation	T-S Observer	FO	PMIO	FUOI
80% *m*	$8.572755 \times 10^{-3}$	$8.572927 \times 10^{-3}$	$8.573402 \times 10^{-3}$	$1.439805 \times 10^{-2}$
80% *v*	$1.550698 \times 10^{-2}$	$1.550715 \times 10^{-2}$	$1.550762 \times 10^{-2}$	$1.511658 \times 10^{-2}$
80% $J_{zz}$	$1.093888 \times 10^{-3}$	$1.093919 \times 10^{-3}$	$1.094219 \times 10^{-3}$	$7.693039 \times 10^{-3}$
90% *m*	$4.511087 \times 10^{-3}$	$4.511258 \times 10^{-3}$	$4.511733 \times 10^{-3}$	$1.068700 \times 10^{-2}$
90% *v*	$8.155572 \times 10^{-3}$	$8.155742 \times 10^{-3}$	$8.156217 \times 10^{-3}$	$1.100316 \times 10^{-2}$
90% $J_{zz}$	$8.681782 \times 10^{-4}$	$8.682074 \times 10^{-4}$	$8.685089 \times 10^{-4}$	$7.746213 \times 10^{-3}$
110% *m*	$4.331730 \times 10^{-3}$	$4.331617 \times 10^{-3}$	$4.331142 \times 10^{-3}$	$8.235821 \times 10^{-3}$
110% *v*	$8.197796 \times 10^{-3}$	$8.197682 \times 10^{-3}$	$8.197209 \times 10^{-3}$	$8.874272 \times 10^{-3}$
110% $J_{zz}$	$8.676059 \times 10^{-4}$	$8.676404 \times 10^{-4}$	$8.679451 \times 10^{-4}$	$7.874509 \times 10^{-3}$
120% *m*	$7.853189 \times 10^{-3}$	$7.853076 \times 10^{-3}$	$7.852602 \times 10^{-3}$	$1.145030 \times 10^{-2}$
120% *v*	$1.569571 \times 10^{-2}$	$1.569560 \times 10^{-2}$	$1.569512 \times 10^{-2}$	$1.255470 \times 10^{-2}$
120% $J_{zz}$	$1.091731 \times 10^{-3}$	$1.091767 \times 10^{-3}$	$1.092059 \times 10^{-3}$	$7.951806 \times 10^{-3}$

**Table 3:** Average of the absolute values of yaw speed estimation error under the influence of parameter variations between 80% and 120% of the nominal values of sprung mass *m*, yaw inertia $J_{zz}$, and vehicle speed *v*.

Variation	T-S Observer	FO	PMIO	FUOI
80% *m*	$2.049318 \times 10^{-2}$	$2.049370 \times 10^{-2}$	$2.049554 \times 10^{-2}$	$1.400406 \times 10^{-1}$
80% *v*	$4.713659 \times 10^{-2}$	$4.713563 \times 10^{-2}$	$4.713424 \times 10^{-2}$	$1.763621 \times 10^{-1}$
80% $J_{zz}$	$6.353814 \times 10^{-3}$	$6.353641 \times 10^{-3}$	$6.355151 \times 10^{-3}$	$1.422198 \times 10^{-1}$
90% *m*	$1.135020 \times 10^{-2}$	$1.135072 \times 10^{-2}$	$1.135255 \times 10^{-2}$	$1.403253 \times 10^{-1}$
90% *v*	$2.314887 \times 10^{-2}$	$2.314790 \times 10^{-2}$	$2.314653 \times 10^{-2}$	$1.470490 \times 10^{-1}$
90% $J_{zz}$	$4.488468 \times 10^{-3}$	$4.488319 \times 10^{-3}$	$4.489798 \times 10^{-3}$	$1.415429 \times 10^{-1}$
110% *m*	$1.094071 \times 10^{-2}$	$1.093985 \times 10^{-2}$	$1.093834 \times 10^{-2}$	$1.417235 \times 10^{-1}$
110% *v*	$1.990221 \times 10^{-2}$	$1.990285 \times 10^{-2}$	$1.990453 \times 10^{-2}$	$1.702555 \times 10^{-1}$
110% $J_{zz}$	$4.461358 \times 10^{-3}$	$4.461129 \times 10^{-3}$	$4.463028 \times 10^{-3}$	$1.403494 \times 10^{-1}$
120% *m*	$1.885237 \times 10^{-2}$	$1.885152 \times 10^{-2}$	$1.885000 \times 10^{-2}$	$1.429076 \times 10^{-1}$
120% *v*	$3.418986 \times 10^{-2}$	$3.419049 \times 10^{-2}$	$3.419217 \times 10^{-2}$	$2.273915 \times 10^{-1}$
120% $J_{zz}$	$6.247438 \times 10^{-3}$	$6.247196 \times 10^{-3}$	$6.248951 \times 10^{-3}$	$1.398695 \times 10^{-1}$

**Table 4:** Average of the absolute values of angular displacement estimation error under the influence of parameter variations between 80% and 120% of the nominal values of sprung mass *m*, yaw inertia $J_{zz}$, and vehicle speed *v*.

Variation	T-S Observer	FO	PMIO	FUOI
80% *m*	$2.596766 \times 10^{-1}$	$4.293318 \times 10^{-1}$	1.792212	$4.045841 \times 10^{-1}$
80% *v*	2.329002	2.300337	2.547281	2.258292
80% $J_{zz}$	$2.520897 \times 10^{-1}$	$4.277754 \times 10^{-1}$	1.803890	$4.114284 \times 10^{-1}$
90% *m*	$2.556199 \times 10^{-1}$	$4.285451 \times 10^{-1}$	1.798431	$4.082100 \times 10^{-1}$
90% *v*	1.425565	1.393410	1.902166	1.341277
90% $J_{zz}$	$2.518906 \times 10^{-1}$	$4.277856 \times 10^{-1}$	1.804117	$4.115698 \times 10^{-1}$
110% *m*	$2.553341 \times 10^{-1}$	$4.270808 \times 10^{-1}$	1.810028	$4.150816 \times 10^{-1}$
110% *v*	1.699205	1.761115	2.894581	1.821777
110% $J_{zz}$	$2.519782 \times 10^{-1}$	$4.278060 \times 10^{-1}$	1.804633	$4.118435 \times 10^{-1}$
120% *m*	$2.588346 \times 10^{-1}$	$4.263980 \times 10^{-1}$	1.815441	$4.183360 \times 10^{-1}$
120% *v*	3.424580	3.492390	4.670917	3.563970
120% $J_{zz}$	$2.521996 \times 10^{-1}$	$4.278163 \times 10^{-1}$	1.804919	$4.119757 \times 10^{-1}$

**Table 5:** Average of the absolute values of road curvature estimation error under the influence of parameter variations between 80% and 120% of the nominal values of sprung mass *m*, yaw inertia $J_{zz}$, and vehicle speed *v*.

Variation	T-S	FO	PMIO	FUOI
80% *m*	$2.783634 \times 10^{-2}$	$4.120203 \times 10^{-2}$	$1.796347 \times 10^{-1}$	$4.032692 \times 10^{-2}$
80% *v*	$9.201958 \times 10^{-2}$	$8.809561 \times 10^{-2}$	$1.766649 \times 10^{-1}$	$1.065191 \times 10^{-1}$
80% $J_{zz}$	$2.700095 \times 10^{-2}$	$4.155340 \times 10^{-2}$	$1.799938 \times 10^{-1}$	$3.939616 \times 10^{-2}$
90% *m*	$2.733717 \times 10^{-2}$	$4.138324 \times 10^{-2}$	$1.798391 \times 10^{-1}$	$3.985879 \times 10^{-2}$
90% *v*	$6.134908 \times 10^{-2}$	$5.740804 \times 10^{-2}$	$1.732794 \times 10^{-1}$	$7.474389 \times 10^{-2}$
90% $J_{zz}$	$2.691988 \times 10^{-2}$	$4.155453 \times 10^{-2}$	$1.800131 \times 10^{-1}$	$3.940483 \times 10^{-2}$
110% *m*	$2.646137 \times 10^{-2}$	$4.171994 \times 10^{-2}$	$1.802215 \times 10^{-1}$	$3.898857 \times 10^{-2}$
110% *v*	$6.177371 \times 10^{-2}$	$7.926158 \times 10^{-2}$	$1.922111 \times 10^{-1}$	$5.686010 \times 10^{-2}$
110% $J_{zz}$	$2.684888 \times 10^{-2}$	$4.155681 \times 10^{-2}$	$1.800584 \times 10^{-1}$	$3.942182 \times 10^{-2}$
120% *m*	$2.616851 \times 10^{-2}$	$4.187663 \times 10^{-2}$	$1.804009 \times 10^{-1}$	$3.858344 \times 10^{-2}$
120% *v*	$1.041368 \times 10^{-1}$	$1.245125 \times 10^{-1}$	$2.153718 \times 10^{-1}$	$9.957376 \times 10^{-2}$
120% $J_{zz}$	$2.683923 \times 10^{-2}$	$4.155796 \times 10^{-2}$	$1.800850 \times 10^{-1}$	$3.943082 \times 10^{-2}$

In summary, the results clearly demonstrate that the proposed T-S observer achieves superior estimation accuracy, faster convergence, and greater robustness than alternative observers. Its ability to reliably track both the system states and unknown inputs under parameter variations confirms its suitability for real-time vehicle state estimation, with the FO observer providing a secondary but less consistent alternative.

### Processor-in-the-Loop (PIL) validation

**Fig. 10:**
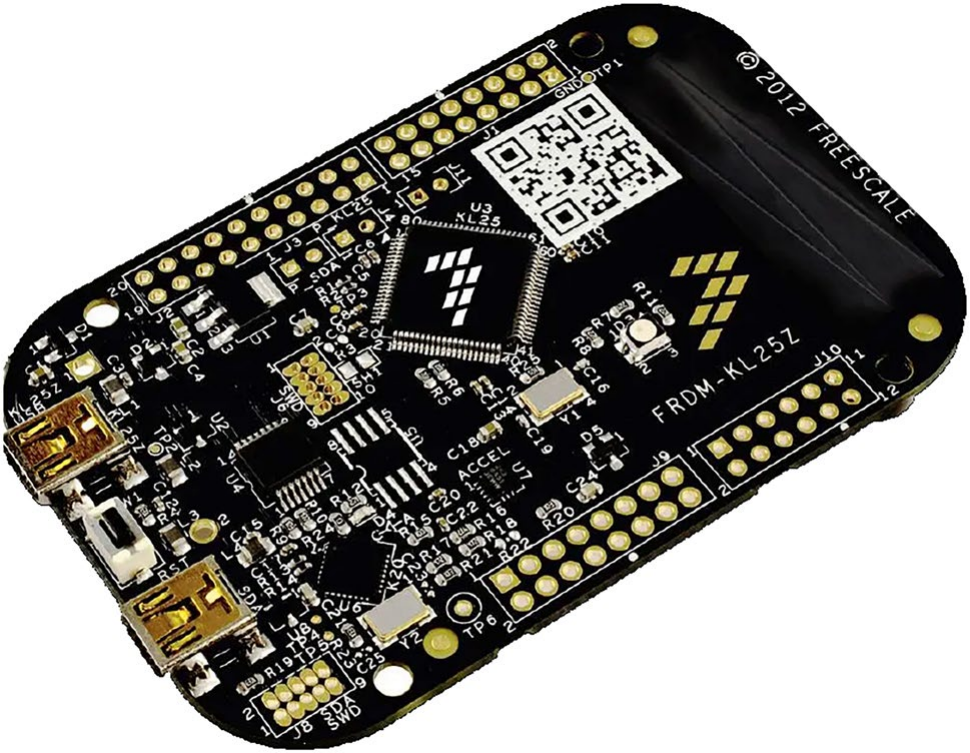
NXP FRDM-KL25Z board.

The NXP FRDM-KL25Z^39^, shown in Fig. 10, is an affordable development platform built around the ARM Cortex-M0+ processor. It integrates 128 KB of flash memory and 16 KB of RAM, making it a compact, yet powerful tool for rapid prototyping. Owing to its balance between low cost and versatility, it has been widely adopted in automotive systems, robotics, and energy-efficient embedded applications.

Processor-in-the-Loop (PIL) is a validation methodology in which automatically generated code from a control or estimation algorithm is deployed to the target microcontroller. This technique ensures that the code runs correctly and efficiently on embedded hardware while maintaining interaction with the simulation environment^40–42^. In doing so, it bridges the gap between simulation and real-time execution, thereby providing confidence in the performance of the algorithm before full deployment.

**Fig. 11:**
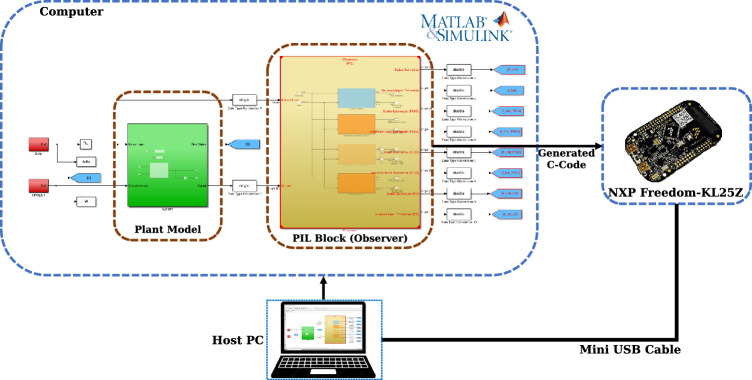
Processor-in-the-Loop (PIL) framework for real-time vehicle state and unknown input estimation.

The functional architecture of the proposed PIL setup is shown in Fig. 11. A vehicle dynamic model executed on a host computer is excited by two categories of inputs: the control signal $\delta (t)$ and an unknown input $\omega (t)$. The model generates measurable outputs $y(t)$, that are sent to the FRDM-KL25Z board through a USB interface.

The KL25Z executes observer-based estimation code generated from Simulink on the embedded side. This algorithm processes the received measurements to reconstruct the system states $\hat{x}(t)$ and estimates the unknown input $\hat{\omega }(t)$. Such a configuration enables the real-time validation of estimation strategies under realistic operating conditions, thereby improving the robustness and accuracy of state and unknown input estimation in advanced automotive and embedded control applications.

**Fig. 12:**
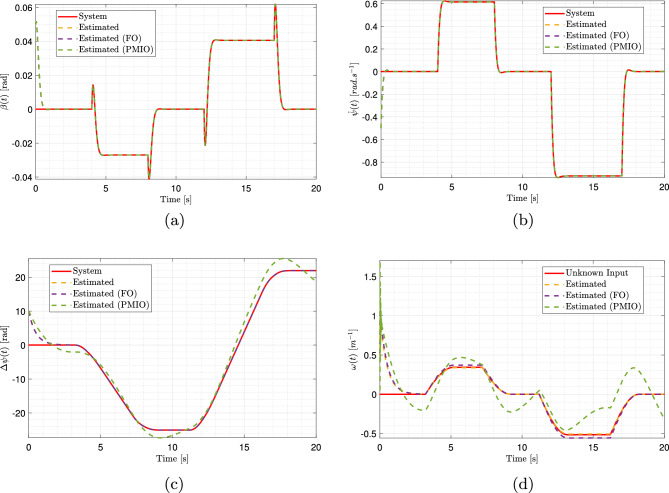
PIL-based estimation of side slip angle, yaw rate, angular displacement, and road curvature with multiple observers. (Solid red: actual system response; dashed orange: proposed observer estimate; dashed purple: FO estimate; dashed green: PMIO estimate).

**Fig. 13:**
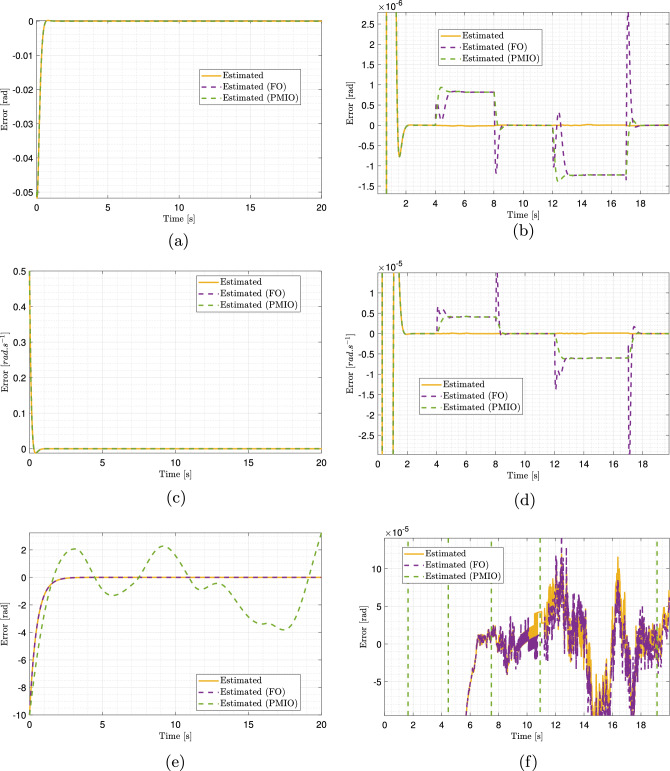
Estimation errors of side slip angle, yaw rate, and angular displacement in PIL with zoomed views on the right. (Solid orange: proposed observer estimate; dashed purple: FO estimate; dashed green: PMIO estimate).

The performance of the proposed T-S observer was further assessed using PIL simulations. Under the same initial conditions, the observer successfully estimated unmeasured system states. Fig. 12a, b, and c illustrate the evolution of the non measured variables. In each case, the estimated trajectories closely followed the actual system responses, with the exception of a noticeable deviation in the PMIO results, particularly in Fig. 12c. The accuracy of the proposed observer is corroborated by Fig. 13, which shows the estimation errors between the true and estimated states. The estimation errors converged asymptotically to zero, thereby confirming both effectiveness and robustness of the proposed observer. In this case, no comparison with FUIO was included, as its performance has not been demonstrated in the MIL validation stage and was therefore excluded from the PIL analysis.

Because the direct verification of the observer’s effectiveness in estimating the system states is not straightforward, the analysis is carried out through estimation errors. Fig. 13a, b, c, and d show the error dynamics for the side-slip angle and yaw rate, respectively. Although the overall plots indicate that all methods achieve errors close to zero, the magnified views clearly highlight the superior accuracy of the proposed observer. Its estimation errors converge more rapidly, remain consistently small, and exhibit high stability with minimal fluctuations, whereas the alternative observers show more pronounced oscillations and larger error peaks, particularly during the transient phases.

The estimation errors for the angular displacement are shown in Fig. 13e and f. While all observers eventually converge toward zero, the zoomed view highlights that the proposed observer achieves the smallest errors and the fastest convergence rate, closely followed by FO. To provide a quantitative comparison, the absolute values of the estimation errors were computed, yielding $0.2492694 \quad rad$ for the proposed observer and $0.2492704 \quad rad$ for FO. These results demonstrate the capability of the proposed observer to provide precise and reliable estimates of the vehicle’s state variables under the simulation, thereby confirming its robustness and effectiveness.

**Fig. 14:**
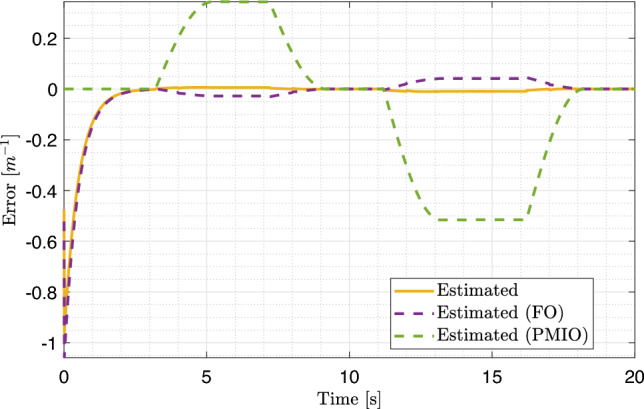
Road curvature estimation errors using different observers in PIL. (Solid orange: proposed observer estimate; dashed purple: FO estimate; dashed green: PMIO estimate).

The estimation of road curvature *w*(*t*), is shown in Fig. 12d. The trajectory of the proposed T-S observer closely follows the actual unknown input, demonstrating a high level of accuracy and fidelity. This near-perfect tracking confirms the observer’s ability to estimate the road curvature with minimal deviation, whereas the alternative approaches, such as the PMIO, exhibit pronounced oscillations and substantial discrepancies from the true value, indicating lower reliability for curvature estimation. The corresponding estimation errors are shown in Fig. 14. The results highlight the rapid response of the proposed observer, characterized by a steep initial slope and fast convergence toward zero error, outperforming alternative approaches. Although FO also achieves low error levels, its convergence is comparatively slower and less stable.

Table 6 presents the quantitative performance of the three observers (proposed T-S observer, FO, and PMIO) for key vehicle states. The metrics included the Root Mean Square Error (RMSE), Steady-State Error (SSE), and convergence time $T_{conv}$. These metrics complement the visual plots by providing a precise numerical assessment of estimation accuracy and speed. Compared to the FO and PMIO observers, the T-S observer provides a more accurate and reliable state estimation, especially for $\Delta \psi (t)$ and $\omega (t)$, where the other observers exhibit significantly larger errors.

**Table 6:** Quantitative error metrics for each observer and variable/.

Observer	Variable	RMSE	SSE	$T_{\text {conv}}$ [s]
T-S Observer	$\beta (t)$	$4.790801\times 10^{-3}$	$2.303179\times 10^{-10}$	2.5
FO	$\beta (t)$	$4.790545\times 10^{-3}$	$6.715777\times 10^{-10}$	2.5
PMIO	$\beta (t)$	$4.790787\times 10^{-3}$	$4.535983\times 10^{-9}$	2.5
T-S Observer	$\dot{\psi }(t)$	$2.694077\times 10^{-2}$	$2.402493\times 10^{-9}$	2.6
FO	$\dot{\psi }(t)$	$2.693925\times 10^{-2}$	$3.595110\times 10^{-9}$	2.6
PMIO	$\dot{\psi }(t)$	$2.694081\times 10^{-2}$	$6.454020\times 10^{-9}$	2.6
T-S Observer	$\Delta \psi (t)$	1.126875	$4.424567\times 10^{-8}$	3.1
FO	$\Delta \psi (t)$	1.148788	$2.024766\times 10^{-1}$	3.1
PMIO	$\Delta \psi (t)$	2.347423	1.779720	4.5
T-S Observer	$\omega (t)$	$1.101480\times 10^{-1}$	$6.373736\times 10^{-5}$	3.2
FO	$\omega (t)$	$1.231379\times 10^{-1}$	$1.045766\times 10^{-4}$	3.2
PMIO	$\omega (t)$	$2.820948\times 10^{-1}$	$5.487260\times 10^{-4}$	4.6

To further validate the proposed observer, both MIL and PIL simulations were conducted. The MIL simulation confirmes the theoretical performance of the observer under ideal conditions, whereas the PIL simulation demonstrated its effectiveness and robustness in a realistic implementation environment. Across both simulation frameworks, the proposed T-S observer consistently achieved near-instantaneous convergence to minimal error, providing faster, more accurate, and more stable road curvature estimation than alternative techniques. These results substantiate the reliability and practical applicability of the proposed observer in real-world vehicle dynamic scenarios.

## Conclusion

This study introduced a T-S fuzzy functional observer designed through LMI optimization to estimate both unmeasured vehicle states (side-slip angle, yaw rate, and angular displacement) and unknown inputs such as road curvature in real time. By relying only on lateral displacement measurements, the proposed approach successfully reconstructed critical vehicle dynamics, providing a practical and computationally efficient alternative to existing methods. Comparative simulations and Processor-in-the-Loop (PIL) validation confirmed its novelty and superiority over FO, PMIO, and FUIO observers, particularly in terms of estimation accuracy, convergence speed, and robustness, thereby demonstrating its potential for integration into advanced driver assistance systems to improve safety. Future work will focus on extending the observer to more complex vehicle dynamics and varied driving conditions, further enhancing its applicability in real-world automotive environments.

## Data Availability

The datasets used and analyzed during the current study are available from the corresponding author upon reasonable request.
